# Smart surgical sutures using soft artificial muscles

**DOI:** 10.1038/s41598-021-01910-2

**Published:** 2021-11-17

**Authors:** Phuoc Thien Phan, Trung Thien Hoang, Mai Thanh Thai, Harrison Low, James Davies, Nigel H. Lovell, Thanh Nho Do

**Affiliations:** Graduate School of Biomedical Engineering, Faculty of Engineering, University of New South Wales (UNSW), Sydney, NSW 2052 Australia

**Keywords:** Engineering, Biomedical engineering, Mechanical engineering

## Abstract

Wound closure with surgical sutures is a critical challenge for flexible endoscopic surgeries. Substantial efforts have been introduced to develop functional and smart surgical sutures to either monitor wound conditions or ease the complexity of knot tying. Although research interests in smart sutures by soft robotic technologies have emerged for years, it is challenging to develop a soft robotic structure that possesses a similar physical structure as conventional sutures while offering a self-tightening knot or anchor to close the wound. This paper introduces a new concept of smart sutures that can be programmed to achieve desired and uniform tension distribution while offering self-tightening knots or automatically deploying secured anchors. The core technology is a soft hydraulic artificial muscle that can be elongated and contracted under applied fluid pressure. Each suture is equipped with a pressure locking mechanism to hold its temporary elongated state and to induce self-shrinking ability. The puncturing and holding force for the smart sutures with anchors are examined. Ex-vivo experiments on fresh porcine stomach and colon demonstrate the usefulness of the new smart sutures. The new approaches are expected to pave the way for the further development of smart sutures that will benefit research, training, and commercialization in the surgical field.

## Introduction

Wound closure is a method to accelerate the healing process of defect tissue at the surgical site^1^. The use of surgical sutures is the most common practice in the medical field as it can promote faster wound healing by connecting tissue with small scar formation. To achieve precise wound closure for the healing process, the ideal suture should possess sufficient mechanical strength and uniform tension distribution as well as high flexibility to enable less suturing complexity during operation^2,3^. However, none of the current commercial sutures can satisfy all these requirements. A variety of materials such as silver, gold, steel wires, animal hair, silk, plant fibers, or tree bark have been used for surgical sutures in the last 4000 years^4^ . Recent advances in functional materials have witnessed the use of synthetic biomaterials such as poly (lactic-co-glycolic acid) or polydioxanone as base materials for surgical sutures. Despite advances, no unique material can meet all types of surgical applications^3–6^. The choice of suture materials for wound closure is closely related to several factors such as layers of tissues, tension, tissue type, surgical access, and time of removal. The use of absorbable and nonabsorbable materials for sutures also depends on the type of wound closure^3,4,7^. Absorbable sutures normally undergo degradation by losing around 50% of their tensile strength within 60 days in the tissues where they are either digested by enzyme or hydrolyzed while nonabsorbable ones exhibit a poor degradation in the body tissues. Some nonabsorbable sutures such as steel sutures for sternal closure or bone can be left permanently in the body.

Advances in minimally invasive surgery such as flexible endoscopy have gained popularity over the past few years. Flexible endoscopic surgeries offer many benefits compared with conventional abdominal open surgery and invasive laparoscopic surgery, including less postoperative pain, faster recovery, and no abdominal skin scars^8^. However, risks of perforation, both accidentally and purposely, have remained a critical challenge for endoscopic procedures^9,10^. Endoscopic submucosal dissection (ESD) is used to remove tumors in the gastrointestinal mucosal layer. ESD has a high risk of perforation (5–10%)^11^ and so requires surgeons with exceptional skills. Endoscopic full-thickness resection (EFTR) is a new method to deal with overgrown tumors in which en bloc tumor removal penetrates the gastrointestinal muscular layer, causing a perforation^11^. Natural orifice transluminal endoscopic surgery (NOTES) is an advanced endoscopic procedure for minimally invasive abdominal surgery where an internal incision in the stomach, colon, or bladder is required for instruments to access the abdominal cavity^9,12^. This internal full-thickness perforation is purposely created and requires closure after finishing the targeted procedure. One of the biggest challenges for the use of surgical sutures for both laparoscopy and endoscopy is the difficulty to close defect tissue effectively with precise sewing and desired tension while maintaining strong knot tying at the surgical site^13,14^. If the suture tension is too high, necrosis of the enclosed and surrounding tissue may occur, leading to unrelieved pain and other unexpected complications for the patients. If the suture tension is too weak, scar tissues with poor mechanical properties may occur, leading to potential hernia and chronic non-healing wounds^3,4^.

Perforation closure is vital to advance the development and enable the full potential of flexible endoscopic surgery, making it an important paradigm shift for minimally invasive endoscopic surgery. Recognizing this prospect, research on an effective solution for perforation closure has proliferated, with a primary focus on the improvement of both surgical sutures and closure devices^15,16^. The research direction can be divided into three main categories. *The first approach* is the use of conventional surgical sutures and combines them with new endoscopic suturing devices. For example, the OverStitch endoscopic suturing system (Apollo Endosurgery, Inc., USA) offers full-thickness sutures for both running and interrupted stitches through a curved needle^17^. The needle punctures and drives the suture through tissue, then two T-tags are deployed to secure closure without the need to tie complex surgical knots. The incisionless operating platform (IOP) (USGI Medical, Inc., USA) has been used for various endoscopic suturing procedures. The platform has multiple tools that can capture and puncture to pass a suture through tissue, then deploy a pair of mesh-shaped expandable tissue anchors to deliver a secure closure^18^. Besides gastrointestinal perforation closure, OverStitch has been used for endoscopic sleeve gastroplasty (ESG) and the IOP used for primary obesity surgery endoluminal (POSE). While weight loss treatments can be performed via capsule endoscopy^19,20^, in general these surgical procedures (ESG and POSE) perform tissue folding to reduce stomach volume and therefore promoting weight loss^21,22^. Another device in the first approach is the robotic suturing system—a master–slave flexible endoscopic system that has two flexible, 5-degree-of-freedom (DOF) robotic arms: a suturing instrument and a grasper^23,24^. The high dexterity robotic arms enable surgeons to create running stitches and tie surgical knots endoscopically via a master console.

*The second approach* is the use of clips which are a type of simple, fast deployable device for endoscopic hemostasis and may be adapted for wound closure without using tendon-like surgical sutures. The size of the hemoclips is small enough to be delivered through endoscope channels. Their two jaws, when deploying, clamp the tissue to stop bleeding^25^. Another technology, over-the-scope clip (OTSC) (Ovesco Endoscopy AG, Germany) is a large nitinol clipping that provides high-force capture to compress the tissue securely for hemostasis and wall lesion closure^26^.

*The third approach* is to improve conventional surgical sutures by incorporating advanced functional materials and new designs with enhanced mechanical properties. Although most surgical sutures for wound closure require secure knot tying with sufficient strength and desired compression to join separated tissues, the creation of a proper knot during the suturing process with sufficient tension is challenging. To avoid knot tying, conventional smooth sutures have been upgraded to barbed sutures that offer self-anchoring to tissues, eliminating the need for a knot^27^. Although barbed mechanisms have been introduced to effectively eliminate the need for complex knot tying and overcome the breakage problem, they still require a high level of suture pretension during the closure procedure before the anchors are deployed^28,29^. A wide range of biomaterials have been used to produce advanced sutures including silk, linen fibers, nylon, polyester, polyurethane, or stainless steel with different surface textures (see^3,4^ for more details). Recent years have revealed an emerging trend towards functional sutures to provide the wound with some additional benefits^3^. Antimicrobial sutures are coated with antimicrobial agents such as triclosan or silver nanoparticles to prevent postoperative infections^7,30^. Furthermore, drug-eluting sutures can release drugs at a specific site to maximize the therapeutic effect^31^. Stem cell seeded sutures can provide biological scaffolds to speed up cell growth and thus accelerate tissue regeneration^32^.

Another emerging tendency for wound closure is the use of programmable sutures. These sutures, a type of filament actuator or sensor, can monitor temperature, pH, or tension of the suture. Shape memory sutures that are made from shape memory polymers (SMPs) can induce self-tightening knots under thermal heated^2,33,34^. Although significant progress has been achieved, this technology requires thermal heating at the suturing site, making them an ill-suited candidate for use in practice. Smart electronic sutures such as the ones developed by Kim et al. are polymer strips with integrated flexible silicone temperature sensors and microheater to monitor wound temperature to support the healing process^6^. Although other electronic sutures with additional sensors to monitor pH, oxygen, temperatures, or wound exudates have been developed^3,6,35^, all of them are not able to adjust tension such as knot tying and this has limited their use in practice.

There is a huge need for a new class of smart surgical sutures that can automatically tighten defect tissues with controlled tension while offering uniform force distribution within the tissue without the need for any human intervention. In this study, we introduce a proof-of-concept of a new smart surgical suture (S^2^ suture) made from a soft hydraulic artificial muscle. The core technology of the S^2^ sutures is the soft, scalable, and flexible tendon-like artificial muscle (STAM) which has been introduced in our previous work^36,37^. Briefly, this soft hydraulic artificial muscle receives input fluid pressure to elongate to the desired length and accumulates potential elastic energy and subsequently generates contraction force, returning to its initial state when releasing the pressure. It can provide high elongation, high length-per-diameter ratio, miniaturization feasibility, and the ability to tune its initial stiffness or generated contraction force. The abundant availability of biocompatible microtubule and micro-coil materials accompanied by the simple fabrication method enables various applications of the STAM for smart sutures.

**Figure 1:**
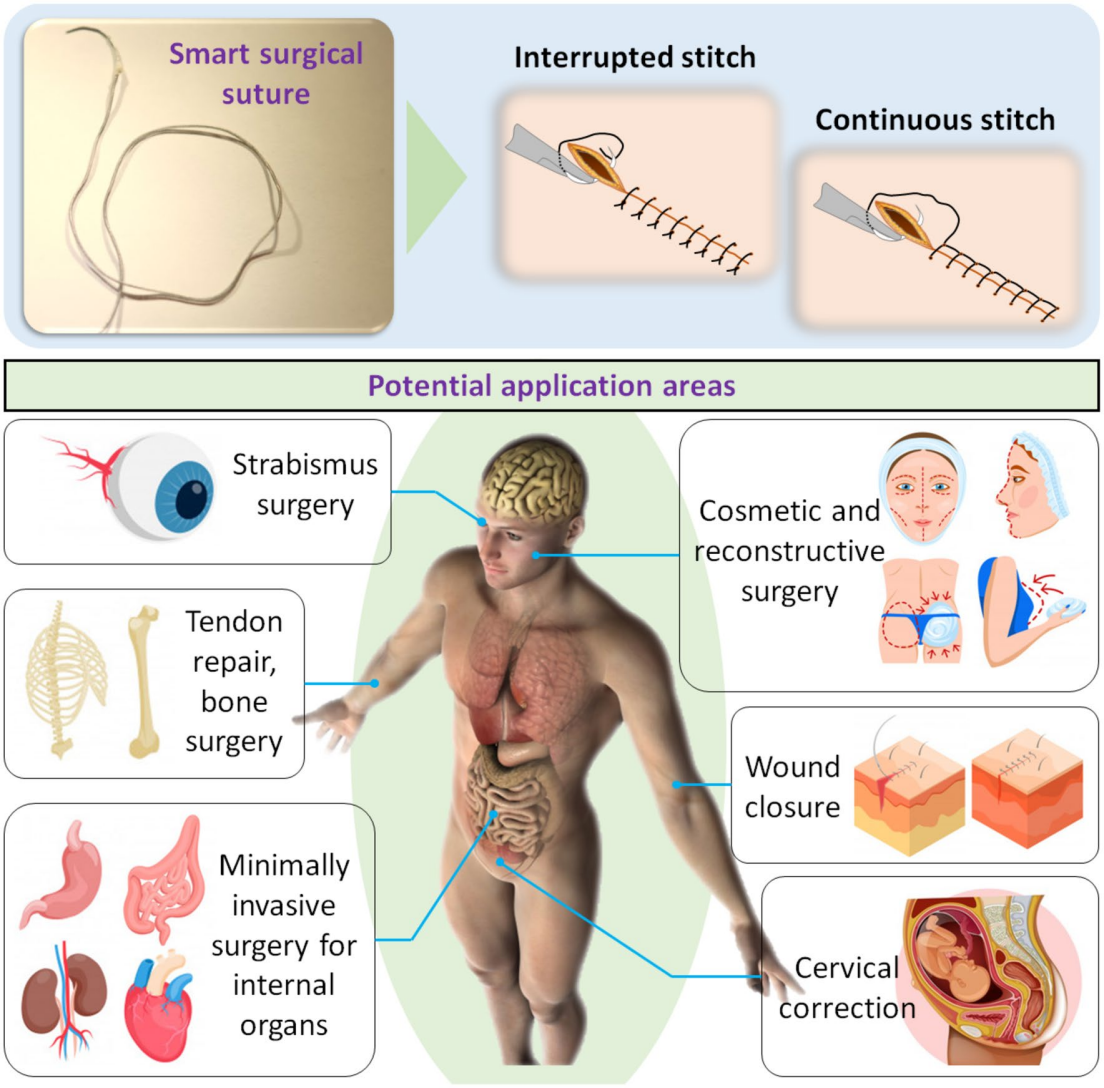
Smart surgical suture with two typical surgical stitches and their potential application areas. The pictures of strabismus surgery, tendon repair, bone surgery, minimally invasive surgery for internal organs, cosmetic and reconstructive surgery, wound closure, and cervical corrections were “Designed by Freepik”.

The S^2^ suture can adjust its length to achieve the desired tension at the time it is fabricated and automatically tighten its knot or deploy anchors to stabilize the suture against the sewed tissues without using any external pulling force, which is normally required in conventional sutures. In addition, our S^2^ sutures are also equipped with different anchors that can effectively secure the defect tissues without the need for using knots, offering flexible choices to meet different demands of wound closure. We also describe the development and manufacture of these new S^2^ sutures, and examine their capability of automatic knot tying and deployable anchors using ex-vivo experiments on fresh porcine stomach and colon. The S^2^ sutures are potential candidates for both interrupted and running (continuous) stitches, thus making them useful for a wide range of applications such as skin wound closure, plastic surgeries, weight loss surgeries, and perforation closure for internal organs (Fig. 1).

## Results

### Smart surgical sutures (S^2^ sutures)

**Figure 2:**
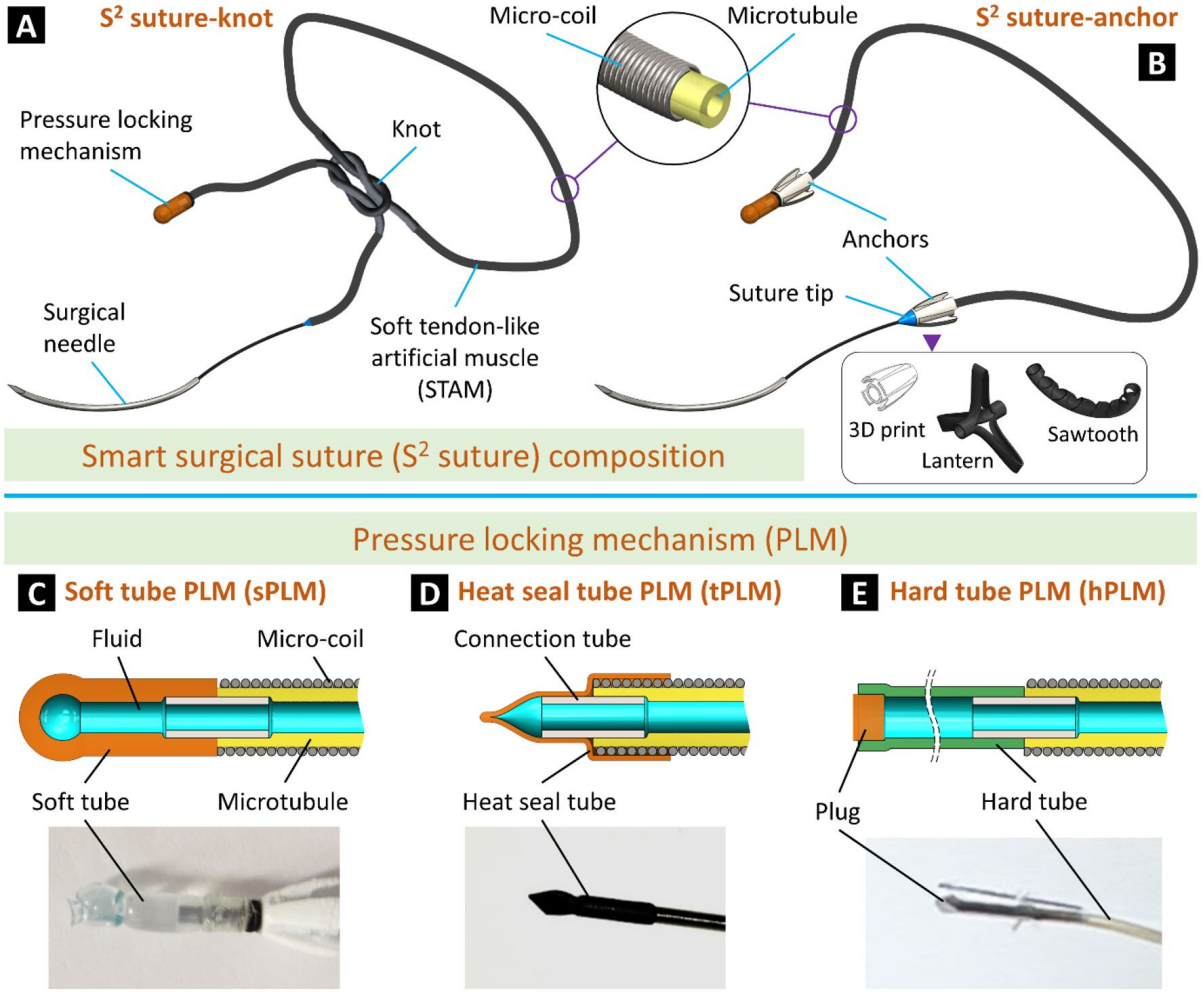
Structure of the smart surgical sutures (S^2^ sutures). (**A**) The S^2^ suture-knot can be knotted as conventional surgical sutures. (**B**) The S^2^ suture-anchor formed by combining the S^2^ suture-knot with three different types of anchors. (**C**–**E**) Design of different pressure locking mechanisms (PLMs) and their prototypes.

In this work, we introduce a new class of soft artificial muscle-driven S^2^ sutures that not only offer effective anchoring functions of both knot tying and barbed anchors but also provide desired and uniform tensile distribution without the need for additional intervention. For ease of comparison, the S^2^ suture with knotting function is named “S^2^ suture-knot” while that with barbed anchors is named “S^2^ suture-anchor.” Typically, the S^2^ suture-knot consists of a soft tendon-like artificial muscle (STAM), a pressure locking mechanism (PLM), and a commercial surgical needle (Fig. 2A). The STAM is a flexible, soft artificial muscle made from a miniature soft silicone tube inserted into a micro-coil so that it can be elongated to store elastic energy upon hydraulic pressurization and exert contraction force when releasing the pressure. Regarding the S^2^ suture-anchor composition, both ends of the STAM are equipped with locking anchors (Fig. 2B). These anchors can be automatically deployed to secure the tissue without the need for a surgical knot, which requires complex manipulation of the closure device. One end of the STAM is connected to a PLM to hold and release its pressure. The other end of the STAM is connected to a cone-shaped suture tip and a curved surgical needle to facilitate the tissue puncture.

We utilize the contraction motion of the STAM after releasing the hydraulic pressure to automatically tighten the suturing knot or deploy anchors when all stitches are made. To do so, the STAM is first pressurized and kept in its stretched state by a PLM. One of the distinguishing features of the STAM compared to conventional sutures is that it provides uniform tension distribution along the artificial muscle body regardless of its length and configuration.

Both the S^2^ suture-knot and S^2^ suture-anchor are equipped with a PLM to hold the inner pressure of the STAM at a predetermined threshold to maintain the desired elongation. After making all stitches, the PLM is cut to release the pressure to shorten the STAM. When producing a STAM, a flexible fluid transmission tube is used to connect the STAM body to a fluid source. While input pressure from the fluid source to the STAM is maintained, the fluid transmission tube is locked and becomes a PLM. We propose three different PLM designs including a soft tube PLM (sPLM, Fig. 2C) made from soft rubber tubing, a heat seal tube PLM (tPLM, Fig. 2D) made from flexible polyethylene terephthalate (PET) tubing, and a hard tube PLM (hPLM, Fig. 2E) made from polytetrafluoroethylene (PTFE) tubing. Three types of PLMs require different locking methods: a simple overhand knot for the sPLM, heat seal effect and reinforced thread for the tPLM, and a cylindrical plug for the hPLM. The sPLM is easier to cut but has a relatively lower pressure threshold and thus smaller suture tension compared to the tPLM and hPLM.

The anchors are responsible for holding separated tissues in place so that surgical knots can be eliminated, releasing surgeons from this arduous task, especially in confined spaces during endoscopic surgeries. We introduce three different designs for the anchors (Fig. 2B): 3D print, lantern, and sawtooth. The 3D printed anchors are made from hard plastic materials by commercial 3D printers. It has a cone shape with four barbs to facilitate one-way tissue puncture and suture locking in the opposite direction. The lantern and sawtooth anchors are flexible plastic hollow tubes with patterned cuts so that they can be deployed to hold the separated tissues once the fluid pressure is released. A tube with longitudinal cuts (spare at two ends) produces a lantern-like shape when lengthwise compressing its two ends. The triangle cuts (sawtooth) create a bending anchor upon deployment where the STAM is shortened after hydraulic depressurization.

**Figure 3:**
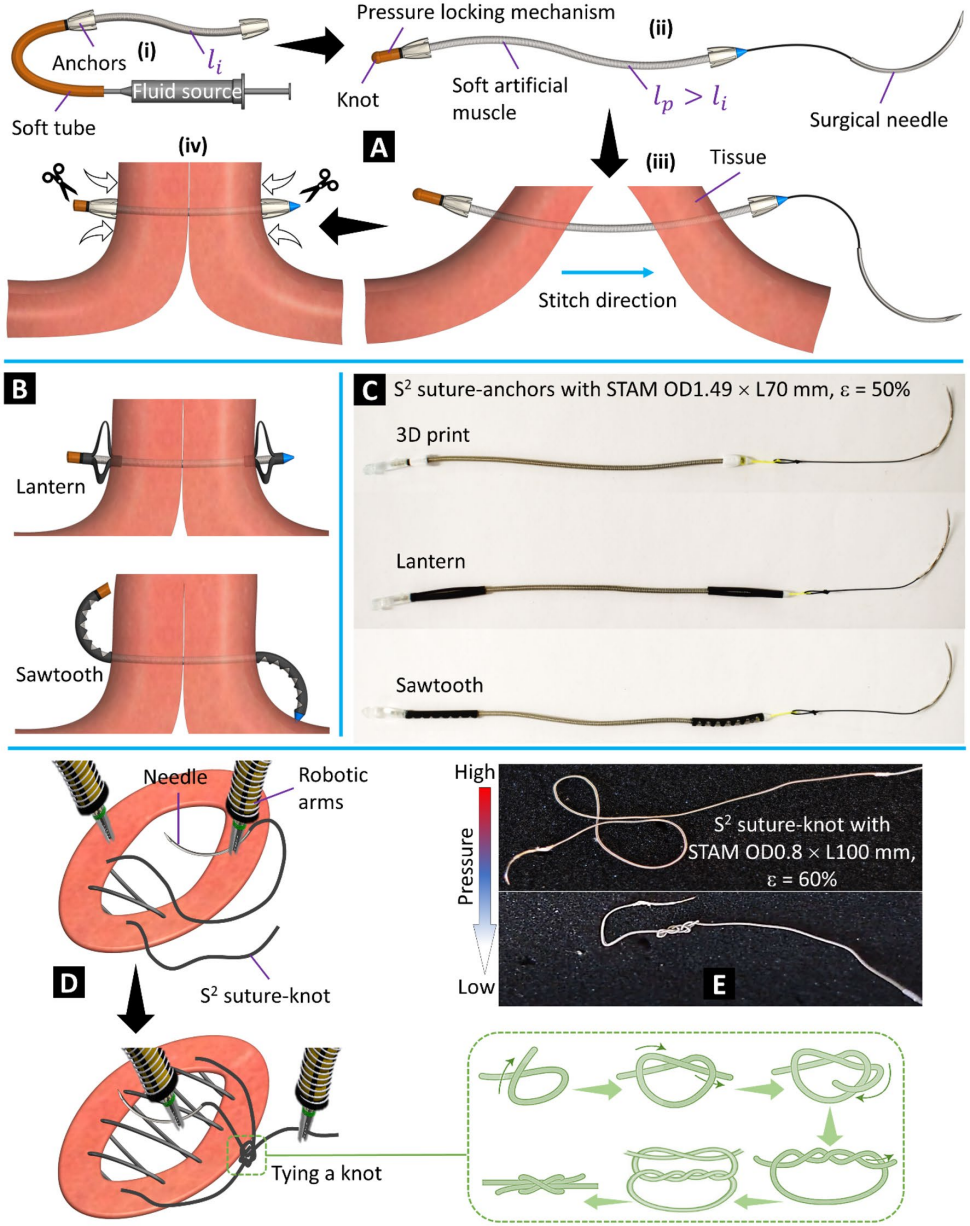
Fabrication, working concept, and prototypes. (**A**) (i) S^2^ suture-anchor with 3D printed anchors at the initial phase (no pressure). (ii) Completed S^2^ suture-anchor with pressurized artificial muscle. (iii) Making stitches through tissue. (iv) Tightening the suture and tissue by releasing pressure. (**B**) Deployment of the lantern and sawtooth anchors after releasing the pressure. (**C**) S^2^ suture-anchor prototypes made from STAM, three types of anchors, and commercial surgical needles (details in Table 1). (**D**) Wound closure procedure using dual surgical robotic arms with continuous stitches of the S^2^ suture-knot. (**E**) An S^2^ suture-knot prototype at high and low pressure.

The fabrication process and working concept of the S^2^ suture-anchor with interrupted stitch are shown in Fig. 3A and the Methods section. The formation of the lantern and sawtooth anchors to secure the tissue is illustrated in Fig. 3B. To validate the new concept, we fabricated three S^2^ suture-anchors with the proposed anchor designs (Fig. 3C). Detailed specifications can be found in Table 1. Each S^2^ suture-anchor consists of a 70-mm-long STAM OD1.49 mm connected to anchors at both ends. Figure 3D illustrates the stitching steps for the S^2^ suture-knot via dual surgical robotic arms and the main procedures to form a secured knot. The suturing procedure of the S^2^ suture-knot with interrupted or continuous stitches is similar to those of conventional surgical sutures where the suture body will form a secured knot to tighten the separated tissues. However, instead of constantly maintaining the suture tension throughout the procedure and tying a tight knot in the case of using conventional surgical sutures, the S^2^ suture-knot implementation allows loose stitches and a loose knot. The suture tension and a tight knot to close and secure the wound will be achieved after releasing the hydraulic pressure. Similar to the S^2^ suture-anchors, we also fabricated two different prototypes for the S^2^ suture-knots (OD1.49 × L100 mm and OD0.8 × L100 mm, details in Table 1) where a surgical needle is directly connected to one end and the other end is equipped with an hPLM. Figure 3E shows an S^2^ suture-knot prototype at high and low input pressure.

**Table 1:** Specifications of S^2^ suture components.

	Micro-coil	Microtubule	
STAM OD1.49	OD 1.49 mm, wire 0.17 mm, maximum strain 416%, stainless steel,	OD 1.19 mm, ID 0.64 mm, maximum strain 786%, silicone rubber, durometer 50A	
STAM OD0.8	OD 0.8 mm, wire 0.065 mm, maximum strain 482%, stainless steel	OD 0.61 mm, ID 0.31 mm, maximum strain 855%, silicone rubber, durometer 50A	

	3D print	Lantern	Sawtooth
Anchor	OD 3.6 mm, L 5 mm, 4 barbs, polylactic acid (PLA +), 3D print	OD 2 mm, ID 1.6 mm, L 20 mm, polyolefin, 3 cuts 120° apart	OD 2 mm, ID 1.6 mm, L 20 mm, polyolefin, cutting angle 80°
Surgical needle	2–0 USP nylon monofilament, reverse cutting curved needle		

### Experimental characterization

#### Evaluation of the STAM capability

**Figure 4:**
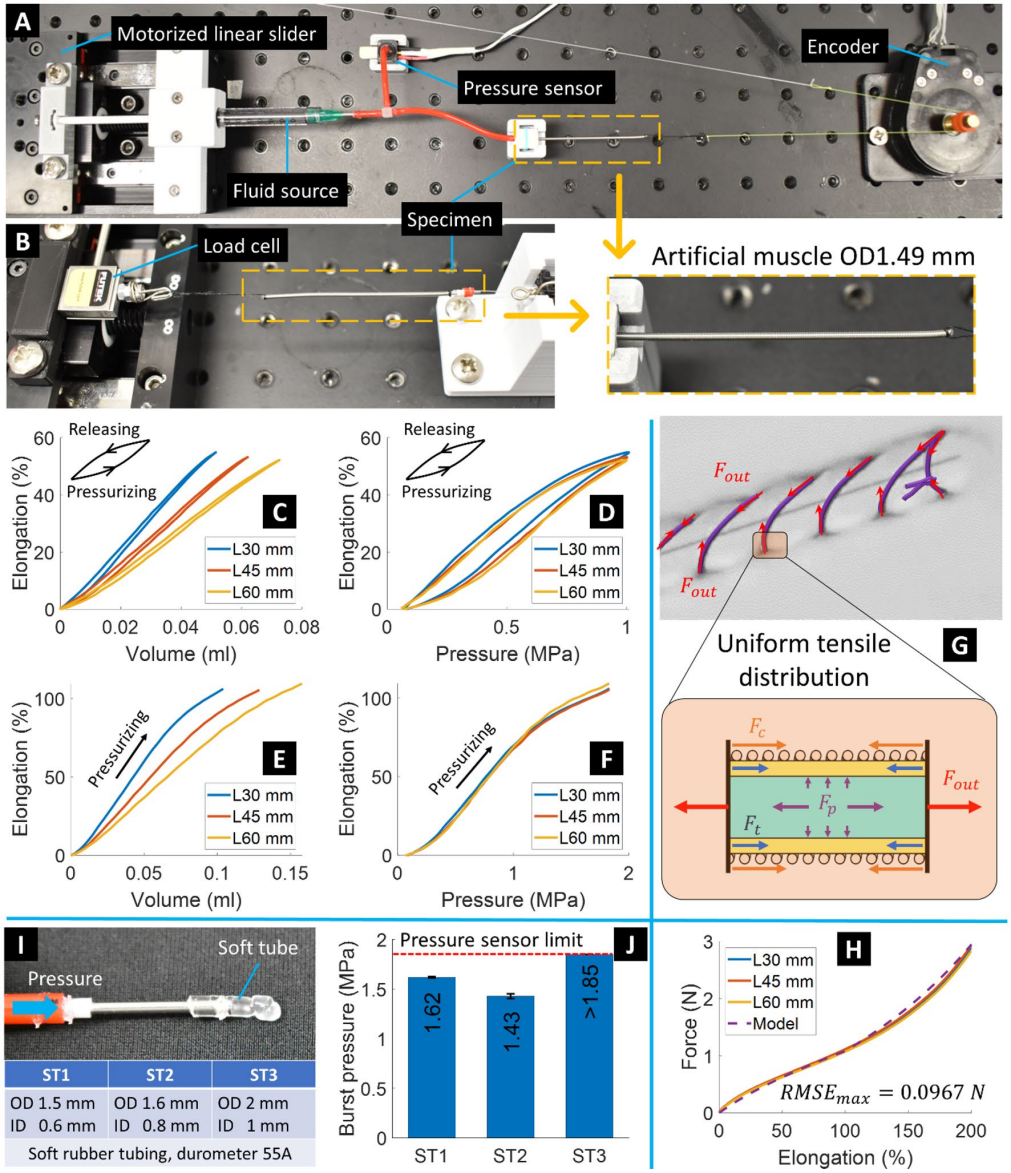
STAM and soft tube characteristics. (**A**) Experimental setup to measure STAM elongation. (**B**) Setup to establish the STAM elongation-force relationship. (**C**, **D**) Hysteresis profiles of input (volume and pressure) and output elongation of three different STAM lengths, when supplying pressure until 1 MPa. (**E**, **F**) Same as C and D but with input pressure reaching pressure sensor limit (1.85 MPa). (**G**) Uniform tension distribution of a small segment of the STAM. (**H**) Elongation-force relationship of three different STAM lengths accompanied by the analytical modeling result (RMSE: root mean square error). (**I**) Setup to measure burst pressure of soft tubes and a table shows specimens’ specifications. (**J**) Burst pressure of three soft tube sizes.

We built a testing platform to characterize the STAM including hysteresis profiles of input volume and pressure versus output elongation, pressure limit, and elongation-force relationship. Details can be found in the Methods section. We produced three STAM specimens with similar specifications (STAM OD1.49, Table 1) but with different lengths of 30, 45, and 60 mm for the experiments. In the elongation experiments, we supplied a 0.2 Hz sinusoidal signal with different amplitudes to the syringe plunger so that each specimen could reach a maximum pressure of 1 MPa. Results (Fig. 4C,D) revealed that output elongation is proportional to both input volume and pressure. Furthermore, different muscle lengths required different input volumes (around 0.05, 0.06, and 0.07 ml for L30, L45, and L60 mm STAM, respectively) to reach 1 MPa. However, this 1 MPa pressure caused similar elongation to three specimens, 54.9% for L30, 53.2% for L45, and 52.2% for L60 mm STAM (standard deviation 1.37%). Hysteresis profiles show a smaller gap between pressurizing and releasing phases in the volume-elongation graph than that of the pressure-elongation graph, depicting that the specimens were more closely responding to volume changes than pressure. Next, we gradually increased input pressure to achieve a higher elongation (over 100%). Results in Fig. 4E,F show similar hysteresis profiles for the pressurizing phase compared to Fig. 4C,D. All three specimens did not burst when reaching the pressure sensor limit (1.85 MPa) and could resume their initial lengths. At 1.85 MPa input pressure, three specimens achieved an elongation of 107 ± 2.1%.

We have investigated the STAM elongation-force relationship by both analytical and experimental approaches (Fig. 4G,H). In the force tests, one end of the STAM was kept static while the other end was pulled by the motorized linear slider (accompanied by a load cell) at a velocity of 10 mm/s until reaching an elongation of 200%. Three specimens with different lengths (30, 45, and 60 mm of STAM OD1.49) were tested, with five trials for each specimen, and mean values plotted. Experimental results show a proportional relation between elongation and force (Fig. 4H) where insignificant deviation can be found between three STAM lengths. They achieved 2.87 ± 0.05 N at 200% elongation.

Force equilibrium of a small STAM section is illustrated in Fig. 4G and described in Eq. (1) whereby the external force *F*_*out*_ and the force *F*_*p*_ caused by hydraulic pressure stretch the section while the micro-coil spring force *F*_*c*_ and the microtubule elastic force *F*_*t*_ tend to return the section to the initial state.

1 $${F}_{out}={F}_{t}+{F}_{c}-{F}_{p}$$

In the context of S^2^ suture implementation where the hydraulic pressure is drained (*F*_*p*_ = 0) to trigger the contraction motion at the end of the suturing procedure, the STAM contraction force will reach the maximum *F*_*out,max*_ = *F*_*t*_ + *F*_*c*_. This equation can be represented in Eq. (2) with detailed explanations presented in reference^36^.

2 $${F}_{out, max}=\alpha E{A}_{t}\left(1-\frac{1}{1+x/{l}_{i}}\right)+{k}_{c}x$$

where *E* and *A*_*t*_ are Young’s modulus and cross-section area of the microtubule, respectively; *k*_*c*_ is the spring constant of the micro-coil; *l*_*i*_ is the STAM initial length; *x* is the STAM displacement when elongating; and stretch ratio *α* is the ratio between the microtubule length and micro-coil length.

In our previous study, the analytical model could effectively capture experimental data until the deflexion point (*ε* = 100%) but showed poor performance at higher elongation. This was because *E* was assumed to be a constant while it was augmented at high strain. This study improves the analytical model given in Eq. (2) by incorporating a dynamic Young’s modulus based on immediate elongation, which is described in Eq. (3). The equation consists of two constituents including the constant value *E*_*0*_ (Young’s modulus at 100% strain) when elongation *ε* ≤ 100% and a simple exponential function with the exponent *ε − *1 when *ε* > 100%.

3 $$E\left(\varepsilon \right)=\left\{\begin{array}{ll}{E}_{0},& \varepsilon \le 100\%\\ {E}_{0}{e}^{\varepsilon -1}, &\varepsilon >100\%\end{array}\right.$$

The dashed line in Fig. 4H represents the analytical model given in Eq. (2) with the following parameters: *α* = 0.9, *A*_*t*_ = 0.8026 mm^2^, *l*_*i*_ = 60 mm, *k*_*c*_ = 0.01 N/mm, *E* follows Eq. (3) with *E*_*0*_ = 1.648 N/mm^2^, *x* = *εl*_*i*_ where *ε* = 0–200%. The analytical model closely followed experimental data the entire elongation range with the maximum root mean square error (RMSE) of 0.0967 N.

#### Burst pressure of the pressure locking mechanism (PLM)

We first examined the burst pressure of the sPLM (soft tube PLM) using a similar setup in Fig. 4A. Detailed processes can be found in the Methods section. Three different sizes of soft rubber tubing (ST1, ST2 and ST3) were tested (Fig. 4I). Mean values and standard deviation of five trials for each tube size are presented in Fig. 4J. The sPLM ST1 and ST2 with a corresponding wall thickness of 0.45 and 0.4 mm could hold a pressure of 1.62 and 1.43 MPa, respectively, before bursting. The burst pressure of the ST3 (wall thickness 0.5 mm) exceeded the pressure sensor limit (1.85 MPa). With the S^2^ suture prototypes in Fig. 3C, if the required elongation is less than 60%, all three sizes of the soft tube can be used as an sPLM. We also tested the burst pressure of the tPLM and hPLM. The results revealed that they could surpass the pressure sensor limit before bursting. Therefore, the PLM and STAM were compatible with each other in terms of input pressure until the threshold of 1.85 MPa.

#### *Anchors’ puncturing and holding force of the S*^*2*^* suture-anchors*

**Figure 5:**
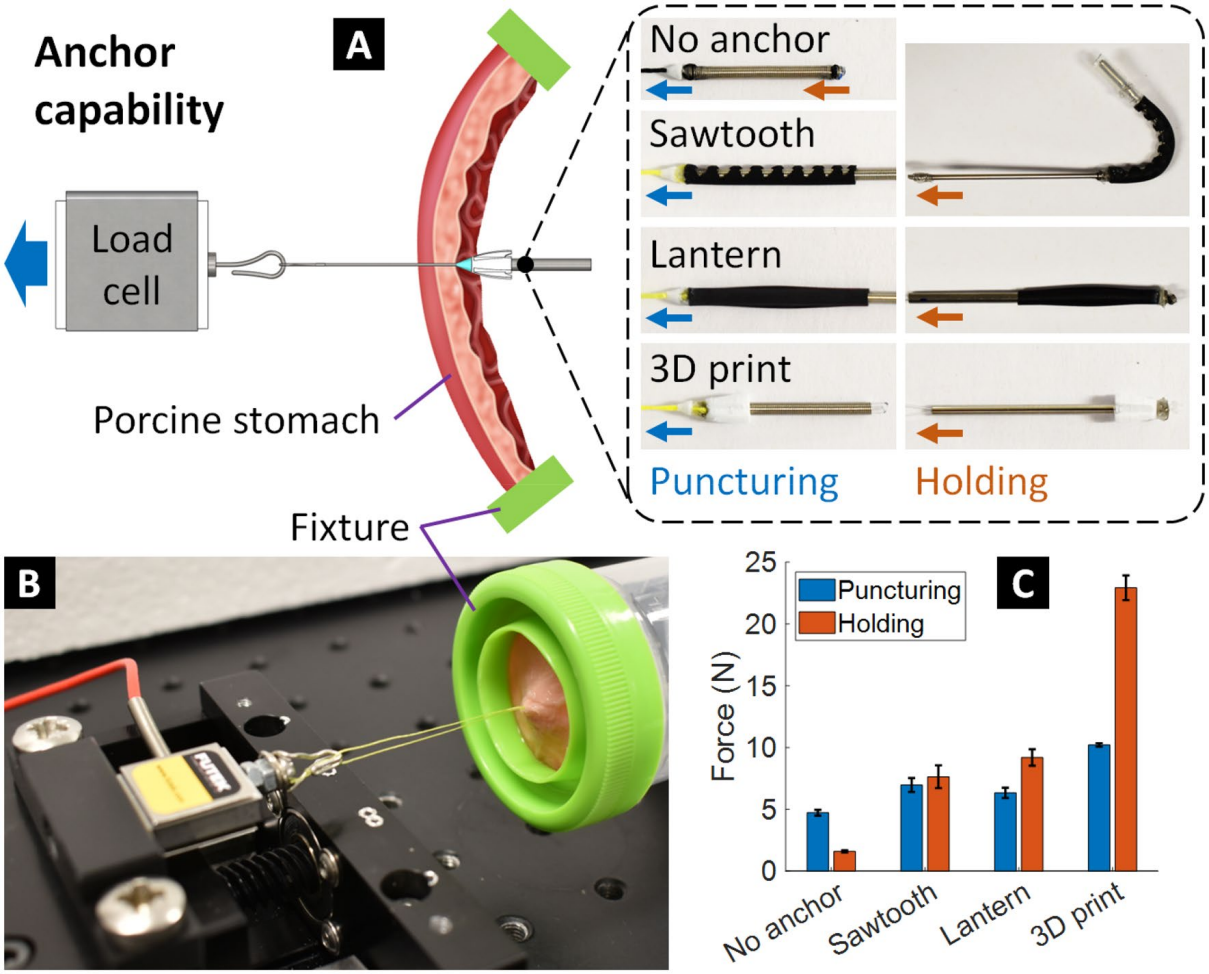
Anchor capability. (**A**) Experimental diagram to measure the puncturing and holding force of different types of anchors through the real porcine stomach tissue (~ 3 mm thickness). (**B**) Actual experimental setup. (**C**) Experimental results.

We also characterized the puncturing and holding force of the S^2^ suture-anchors. We created three different designs for the anchor specimens: sawtooth design, lantern design, and 3D printing design where their specifications are shown in Table 1. We also fabricated an S^2^ suture without anchors (a single STAM only) as a baseline comparison. The experimental diagram and actual setup can be found in Fig. 5A,B where a motorized linear slider, accompanied by a load cell, pulls the anchor specimens through a real porcine stomach tissue (~ 3 mm thickness, Coles supermarket, Sydney, Australia). The tip of each specimen is equipped with a miniature cone-shaped 3D printed block (see the S^2^ suture-anchor design) to ease the puncturing. The same procedure was applied for holding force characterization. However, the anchor specimens have been deployed and switched to the opposite direction.

In contrast, the S^2^ suture without anchor could provide both puncturing and holding force in one pulling motion. Mean values and standard deviation of five trials for each specimen (Fig. 5C) revealed that the S^2^ sutures with anchors have a larger puncturing force and a much higher holding force compared to the baseline (S^2^ suture without anchor). While the specimen without anchor required around 4.7 N to penetrate the porcine stomach, the sawtooth, lantern and 3D printed anchors needed around 7 N (1.49 times larger), 6.3 N (1.34 times larger), and 10.2 N (2.17 times larger), respectively. Regarding the holding force, the specimen without anchor could hold only around 1.6 N whereas the sawtooth and lantern anchors could achieve holding forces of 7.6 N (4.75 times larger) and 9.2 N (5.75 times larger), respectively. Impressively, the 3D printed anchor specimen could hold 22.9 N (14.3 times larger) before perforating the tissue.

#### *Knot security and self-tightening capability of the S*^*2*^* suture-knot*

For wound closure during surgical procedures, tying knots with sutures is an essential component of maintaining tissue opposition where the security of a knot is crucial to hold separated tissues for promoting tissue healing. In this section, we will validate our hypothesis that the developed S^2^ suture-knot could be applied loosely in its temporary shape during the suturing process. Once the temporary knot is created, the hydraulic pressure will be reduced so that the suture will be shrunk to automatically tighten the knot with a pre-determined tension. We performed a set of experiments to test the feasibility of these concepts for our S^2^ suture-knots including the ability to automatically tighten a knot with different loops and the knot security such as knots untying, or suture slipping from the clamps.

**Figure 6:**
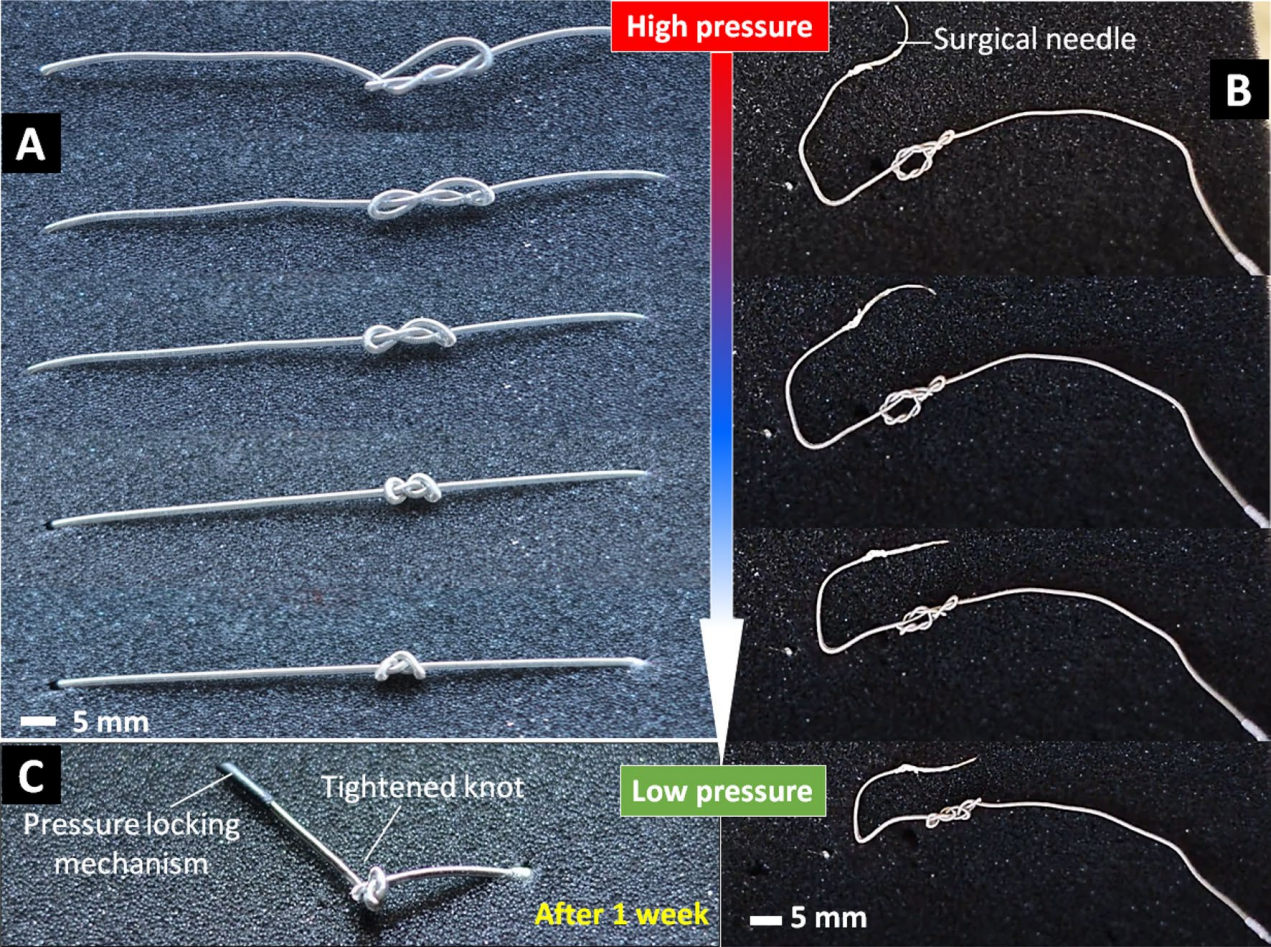
Self-tightening capability and knot security of the S^2^ suture-knot. (**A**) A prototype (OD1.49 × L70 mm) is pressurized to 100% elongation and tied a loose knot with both ends are fixed. The knot is tightened when reducing input pressure. (**B**) Similar to A but with a prototype OD0.8 × L100 mm and both ends are set free. (**C**) Stability of the tightened knots after 1 week. These experiments are available in the Supporting Video.

As an illustration, we fabricated two different prototypes of the S^2^ suture-knots (STAM OD1.49 and STAM OD0.8, Table 1). We elongated the S^2^ suture-knot (OD1.49 × L70 mm) to about 100% elongation, formed a loose knot (Fig. 6A) and fixed both ends. We then withdrew the fluid (water) by slowly reducing the pressure via a miniature syringe. In this experiment, we did not perform the tension measurement of the S^2^ suture-knot, but we instead examined its self-tightening capability and knot security. Figure 6A shows how the suture knot is formed and automatically tightened when the STAM is shortened. We also conducted other experiments to examine the self-tightening capability of the knot and its security in the case when both ends of the suture were set free. Figure 6B shows the process of forming a tightened knot of an S^2^ suture-knot (OD0.8 × L100 mm) once the fluid pressure is reduced. The results from Fig. 6 show that our S^2^ sutures were able to self-shrink and tighten their knots with the desired tension (corresponding to pre-determined elongation, Fig. 4H). The secured knot was well maintained without occurring any failures such as slipping open or being untied after 1 week (Fig. 6C). These results also confirmed our hypotheses that the S^2^ sutures could automatically tighten their knot without using any external human intervention.

### Ex-vivo experimental validation

A major challenge for most wound closure procedures is the maintenance of high pulling force and uniform tension for the suture during the surgical procedure. This section will demonstrate proof-of-concept for the use of our S^2^ sutures with uniform tensile distribution without the requirement of high pulling force in several surgical procedures. They include full-thickness defect closure or perforation closure, gastric plication for weight loss surgery, and proof-of-concept of cervical correction of the cervix. All procedures were performed on a fresh porcine colon and stomach (Coles Supermarkets, Sydney, Australia).

#### *Perforation closure with the S*^*2*^* suture-anchors*

**Figure 7:**
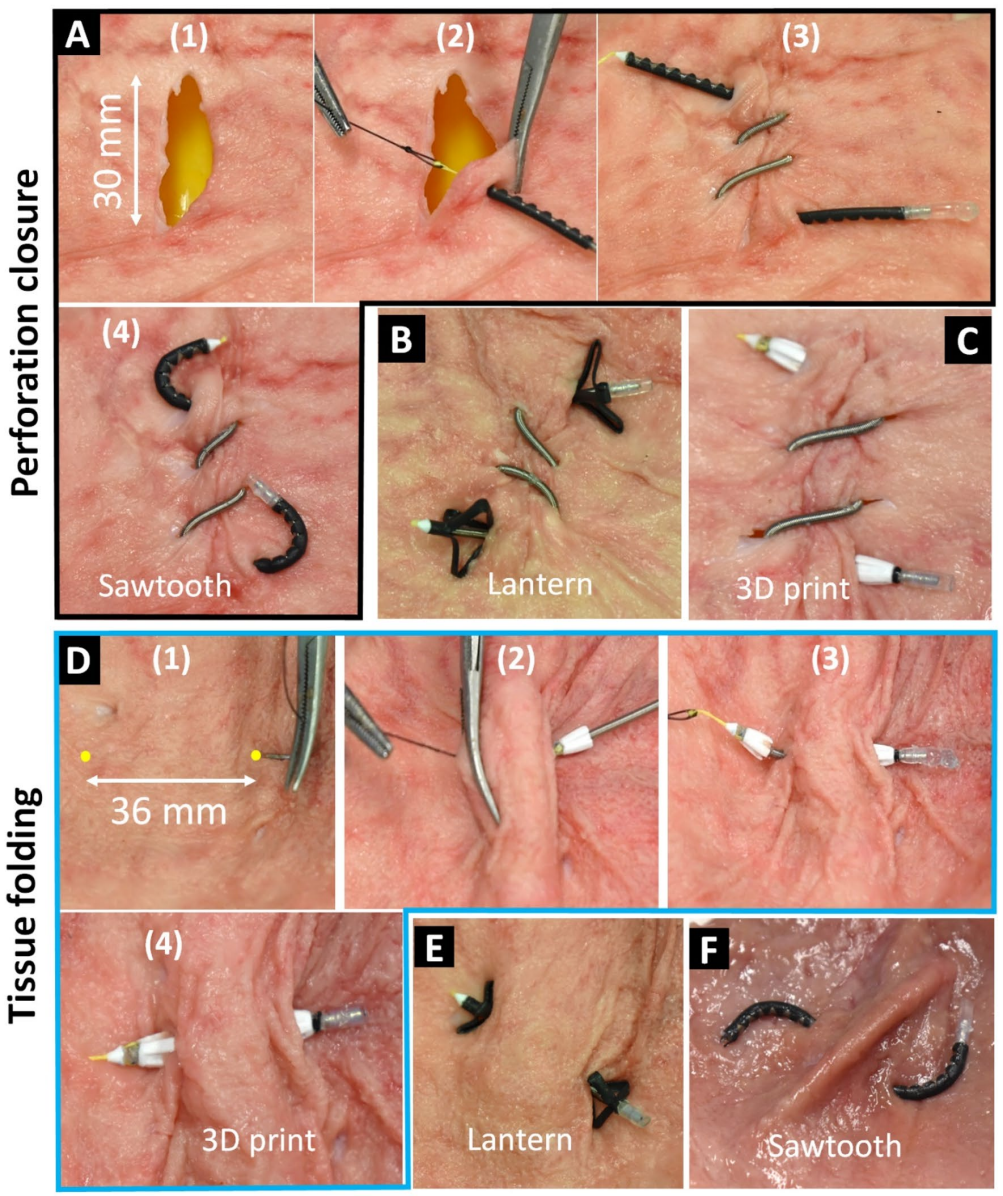
Perforation closure and tissue folding procedure with the S^2^ suture-anchors. (**A**) Perforation closure procedure with six running stitches by sawtooth anchor suture. (**B**, **C**) The same procedure applies to the lantern and 3D printed anchor sutures, respectively. (**D**) Tissue folding procedure (weight loss surgery) by 3D printed anchor suture. (**E**, **F**) The same procedure applies to the lantern and sawtooth anchor sutures, respectively.

We fabricated three prototypes of the S^2^ suture-anchors using the same size STAM (OD1.49 × L70 mm). Each STAM was hydraulically pressurized and locked at 50% elongation and equipped with different anchors and surgical needles (Fig. 3C). We first created a straight 30-mm-long perforation on the fresh porcine stomach (Fig. 7A). Next, we manually made six running stitches by the S^2^ suture around the perforation to close the wound using two pairs of surgical tweezers. Each stitch was made by puncturing the tissue with a surgical needle followed by the STAM body. We then cut the pressure locking mechanism to release the inner pressure which automatically deployed the anchors and then tighten the separated tissues. The entire perforation closure procedures for the S^2^ suture-anchors with sawtooth are demonstrated in Fig. 7A and the Supporting Video. We also repeated the same procedures for the S^2^ suture-anchors with lantern and 3D printed anchors. The results are shown in Fig. 7B,C. The successful closures for the perforation indicate that all three S^2^ suture-anchor prototypes were working as desired and could successfully achieve the required perforation closure requirement.

#### *Tissue folding with the S*^*2*^* suture-anchors*

Gastric plication is a minimally invasive weight-loss surgical technique that reduces the size of the stomach to promote weight loss^21,22^. This technique does not have any implanted devices such as gastric banding or involve any removal of portions of the stomach. The tissue folding technique or gastric plication is a weight-loss surgical technique that normally requires the use of surgical sutures where high tension is required to tie a knot and to maintain a sufficient level of force to fold the tissue. In this technique, each fold is completed by making and tightening a bilateral stitch. To demonstrate the capability of our S^2^ suture, we produced another three prototypes of the S^2^ suture-anchors (similar to those in Fig. 3C but with shorter initial lengths of 30 mm). We designed the suture in a way that we can achieve a pair of stitches with a distance of around 36 mm that can fully fold the porcine stomach tissue (Fig. 7D). After double-puncturing (in and out) the tissue by the surgical needle, we pulled one anchor of the S^2^ suture through the double-wall thickness tissue. We then cut the pressure locking mechanism to shorten the STAM length which subsequently formed a stomach fold. The surgical needle was then cut and removed. Figure 7D shows the entire tissue folding procedure using the 3D printed anchor suture. The tissue folding results of the lantern and sawtooth anchor are presented in Fig. 7E,F.

#### *Perforation closure with the S*^*2*^* suture-knots*

**Figure 8:**
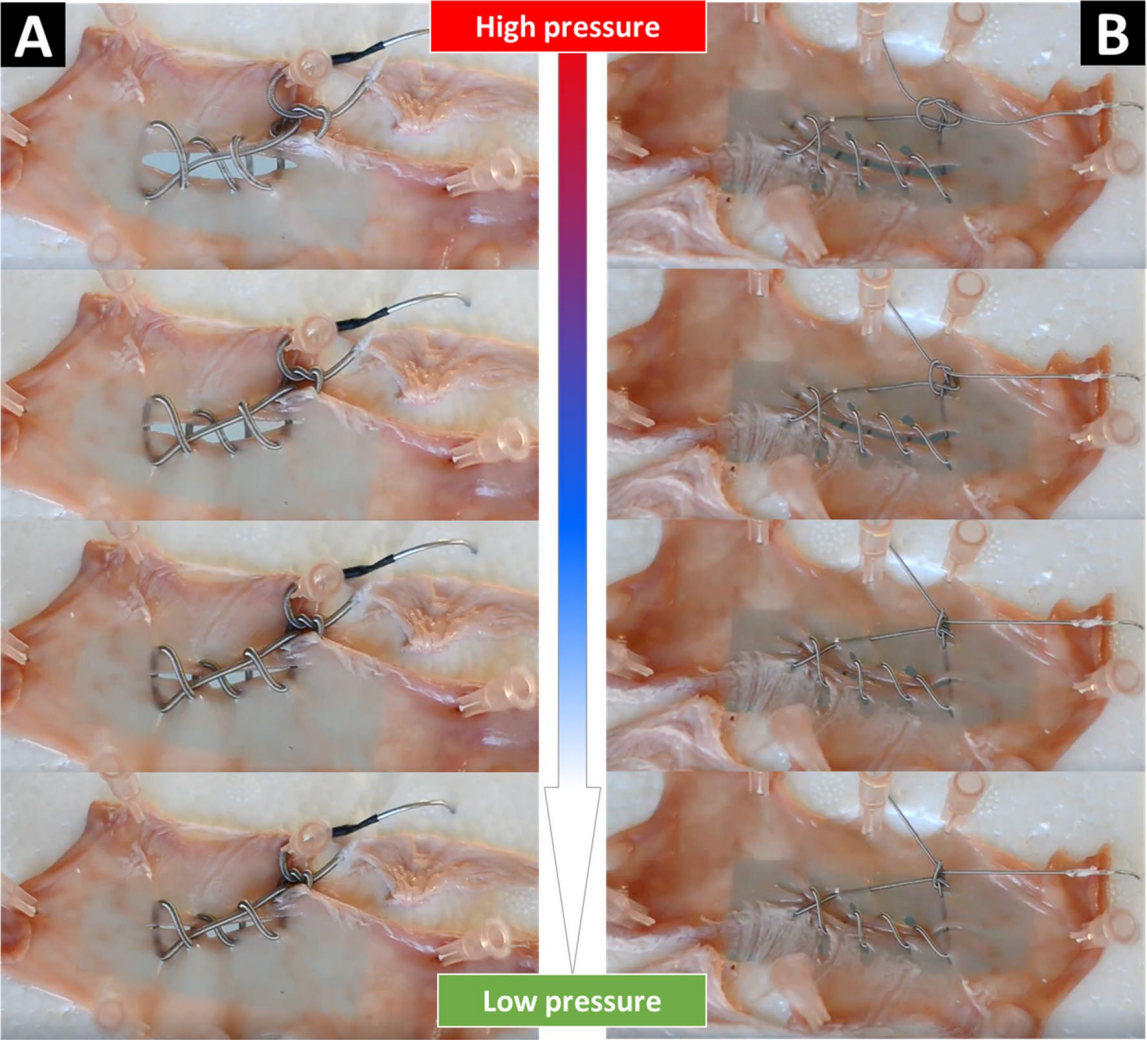
Perforation closure with the S^2^ suture-knots on a fresh porcine colon. (**A**) Results for the S^2^ suture-knot OD1.49 × L70 mm. (**B**) results for the S^2^ suture-knot OD0.8 × L100 mm. These demonstrations are available in the Supporting Video.

We also created two different prototypes of the S^2^ suture-knots where their STAM had a diameter of 1.49 mm and 0.8 mm, respectively. Details of the characterization process can be found in the Methods section. Figure 8 shows the perforation closure results for the two prototypes of S^2^ suture-knots on a fresh porcine colon. During the experiments, we observed that the S^2^ suture-knots with the smaller diameter of STAM formed a secure and self-tightening knot more quickly than that of the larger STAM once the fluid pressure was reduced. This can be explained by the effect of the frictional surface of the outer micro-coil, its stiffness, and flexibility. In clinical practice, the lamination of soft biocompatible silicone or other polymers or coatings on the outer surface of the suture body is strongly recommended. This will contribute to a reduction in the surface friction of the STAM. In addition, a higher elongation of the STAM could promote the security of the knot tying.

#### *S*^*2*^* suture-knots for cerclage correction of the cervix*

**Figure 9:**
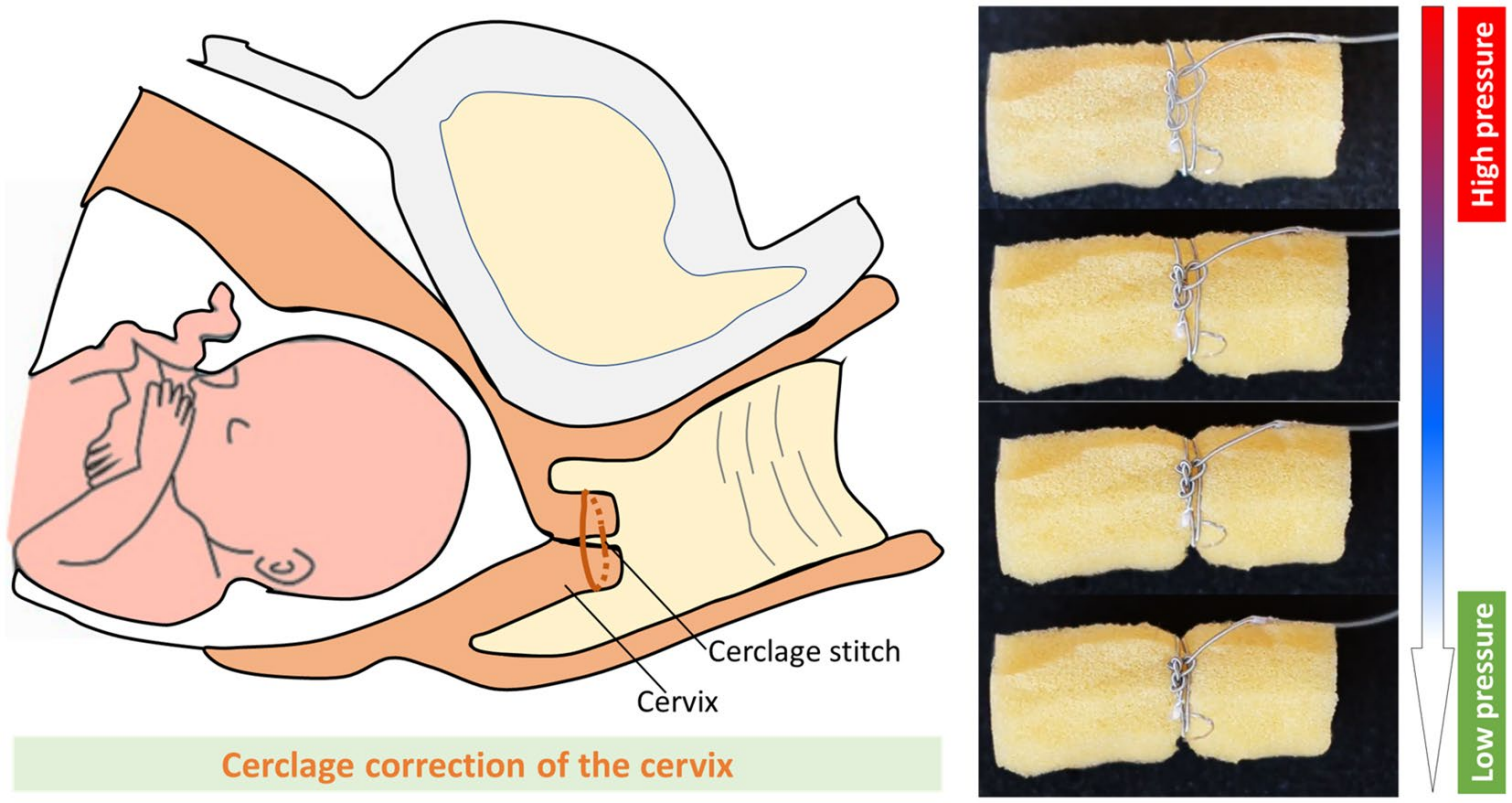
Proof of concept for the cerclage correction of the cervix with the S^2^ suture-knot. The prototype is pressurized to 60% elongation, wrapped around a soft foam, tied a knot, and finally released the fluid pressure to self-tighten and secure the knot.

One of the main reasons for early opening of the cervix during childbearing is cervical insufficiency, meaning that the cervix is weak and unable to remain closed until the date of birth delivery^38,39^. The correction of the cervix mainly involves the use of sutures to wrap around the cervix during pregnancy. Despite advances in the progress of medical technologies in the last few decades, the use of surgical sutures to temporarily treat recurrent pregnancy loss or premature birth still remains a major challenge in surgical procedures for cerclage correction. We demonstrate here that our S^2^ suture-knot could be used as a potential candidate to support the cerclage correction for the cervix without requiring any external pulling force while providing uniform tension to close the cervix. As a proof of concept, we fabricated a prototype of the S^2^ suture-knot (OD0.8 × L100 mm). The S^2^ suture-knot was pressurized to 60% elongation before wrapping around a soft cylindrical foam simulated as the human cervix. Once the S^2^ suture-knot was fully wrapped around the soft foam and a loose knot was successfully created, we then quickly released the hydraulic pressure inside the STAM to self-tighten the knot. Figure 9 shows the experimental results for the proof-of-concept cerclage correction for the cervix. We visually observed a steady radial deformation of the soft foam under the S^2^ suture-knot compression force. In addition, it could form a self-tightening knot once the fluid pressure was withdrawn.

## Discussion and conclusion

Surgical sutures with self-tightening capability and uniform tensile distribution are highly desired in many surgical applications. The right tension in surgical sutures is an important factor to promote optimal tissue healing. A strong tension can restrict the blood flow and therefore create necrosis while a weak tension will result in the opening of incisions and thus the wound not healing properly. Tying a knot or providing high tension for sutures either in interrupted or continuous stitches to close an open wound or to fold a tissue area during endoscopic surgery has long been a critical challenge for surgeons and suturing devices. Despite significant advances in robotic technologies for perforation closure, it is also critical and particularly complicated to precisely control the suture tension and automatically tighten the knot or anchor to close the wound lips with the right stress. This has been a major concern for surgeons for decades^4^.

We have demonstrated proof-of-concept of new S^2^ sutures that upon triggering can automatically tighten a loose knot or deploy anchors to close separated tissues. Although soft robotic technologies have emerged recently^40–48^ to the best of our knowledge, there are no miniature soft fluid-driven actuators (diameter less than 0.8 mm, length longer than 1000 mm) that can be used as a surgical suture to perform the function of self-tightening a knot and anchoring as we have presented. One of the key components of our S^2^ suture is a STAM that can elongate back and forth to induce uniform contraction force along its body under applied input hydraulic (water) pressure. A single STAM (OD1.49 mm) could hold a pressure up to 1.85 MPa without rupture and generate 107% elongation. At 200% elongation, it could produce a contraction force of 2.87 N. The STAM tension force is a combination of the micro-coil linear spring force and the microtubule nonlinear elastic force. These two forces are influenced by material properties and strain. In the case of uniformity of the micro-coil and microtubule materials, every small section like those in Fig. 4G can represent the force distribution of the whole STAM. When releasing input pressure, every STAM section experiences similar locally and simultaneously contraction motion to discharge elastic energy, leading to uniform tensile distribution of the whole S^2^ suture.

We also introduced different pressure locking mechanisms (PLMs) including sPLM, tPLM, and hPLM to maintain the elongated state of the S^2^ sutures during the suturing procedure and could be cut afterward to tighten the separated tissues. To secure the tissue, we also created three different types of anchors which relieve the surgeons from tying a complex surgical knot. Three designs for the anchors (3D printing, lantern shape, and sawtooth shape) have been designed to minimize puncturing force in one way and obstruct in the opposite direction which significantly raises the holding force. The sawtooth, lantern, and 3D printed anchor of the S^2^ suture-anchors required 7 N, 6.3 N, and 10.2 N, respectively, to penetrate a 3 mm thick porcine stomach tissue. Their corresponding holding forces were 7.6 N, 9.2 N, and 22.9 N. It is noted that all three anchor specimens had larger outer diameters compared with a single STAM so that they required a higher force to puncture the tissue. The higher puncturing force compared to holding force from the STAM with no anchors can be solved by using a smooth connector or a larger surgical needle. The sawtooth and lantern anchors required an equivalent puncturing force as they were made from the same flexible tube. However, after being deployed, the lantern-shaped anchor provided a larger holding force than the bending-shape of the sawtooth anchor. The rigid 3D printed anchor had the largest outer diameter causing the largest puncturing force amongst the testing specimens. On the other hand, the use of rigid materials with large diameter and barbs gave the 3D printed anchor a significant holding force. A suture tip and a commercial surgical needle were employed as a guide to facilitate the puncturing step.

All three S^2^ suture-anchors have been successfully demonstrated functionality-wise in ex-vivo experiments where they could close a 30-mm-long perforation of a fresh porcine stomach by six running stitches. Also, the S^2^ suture-anchors could create a tissue fold by tightening a bilateral stitch. Multiple tissue folds may be required in order to achieve the desired stomach volume reduction for weight loss control applications. It is noted that the perforation closure and tissue folding procedures were performed under an open surgery-like setting with no space restrictions and without dynamic movements of the tissue. Although we have not quantitatively evaluated the possible effects of high burst pressure on tissues yet, we hypothesize that it is unlikely that the burst pressure from our smart sutures will cause any harm to surrounding tissues. In our approach, the sutures carry a very small and fixed amount of fluid volume (< 0.1 ml). When cutting the pressure locking mechanism, the inner pressure of the sutures drops rapidly and immediately, causing an instantaneous fluid jet to firstly hit the cutting instrument and dissipate smoothly and gently to the surrounding environment. In ex-vivo experiments, we also observed that there was no fluid jet coming to contact directly with the porcine tissues and therefore the harmfulness to the surrounding tissues are unlikely. However, we would recommend that more experiments to study the effect of high burst pressure on surrounding tissues should be carried out in future works before implementing the smart sutures in preclinical and clinical trials.

We also developed the S^2^ suture-knot that is an S^2^ suture without anchors. The S^2^ suture-knots can be used as conventional surgical sutures. However, they could be automatically tightened, securing their knot without using any external human intervention or pulling force. In addition, the tied knot was able to hold its secured shape after 1 week without occurring any failures caused by slip or opening. To further validate the usefulness of the S^2^ suture-knots for wound closure applications where knot tying is highly desired, we performed ex-vivo perforation closure experiments on the fresh porcine colon. We also conducted a proof-of-concept experiment to demonstrate the use of our S^2^ suture-knot for a complex surgical procedure—the cerclage correction of the cervix. All results confirmed that our new S^2^ suture-knots were able to automatically tighten and secure the target tissues at desired suture tension, which is challenging for existing suture technologies.

It is noted that the current S^2^ suture size is around 0.8 mm in diameter, which is still considered large compared to commercial sutures. In future work, smaller sizes of the suture (< 0.5 mm in diameter) will be fabricated. In addition, all prototypes in this paper use a stretch ratio *α* = 0.9–1 which is not an optimal design. We previously demonstrated that a reduction of the stretch ratio will increase its stored elastic energy and thus exert a higher contraction force. For the next phase of the project, we will also re-design the current STAM with relevant finite element analyses (FEAs) via COMSOL Multiphysics ( COMSOL Inc., USA) in order to determine the desired parameters towards the achievement of the optimal smart suture. For further development, the S^2^ suture-anchors can be also improved to some extent. *Firstly,* the S^2^ suture-anchors have a relatively large size and larger puncturing force compared with conventional sutures, restricting their use in practice. Therefore, miniaturization of the anchor sizes is crucial for it to be used in clinical suturing procedures. *Secondly,* the current S^2^ suture-anchors have a predefined distance between two anchors. As a result, the surgeons need to evaluate the perforation before choosing a certain length of S^2^ suture. Although the developed S^2^ sutures could not precisely control a precise value of the final tension, however they are able to provide a uniform tension distribution to close the wound that is impossible for existing sutures. As discussed in the introduction section, the uniform tension distribution for surgical sutures will facilitate the need of applying high tension for wound closure which is challenging in existing closure procedures including endoscopic surgery. In this paper, the S^2^ sutures were designed in a way that they can provide a tension force within a desired range which is not too loose or too tight. Before suturing procedure, the S^2^ sutures were pressurized to a certain initial elongation which corresponds to final tension once the pressure is released and the surgeons will perform closure procedure with stitching where a high pre-tension (which is highly desired in current surgical sutures) for the smart suture is not required before securing a know. It is noted the precise final tension of the S^2^ sutures heavily relied on surgeons’ stitching skills. Therefore, future work should focus on a mechanism to adjust the anchor distance dynamically on-site to actively control the suture tension.

Both types of the S^2^ sutures were made from non-absorbable materials such as stainless steel for the outer coil and silicone elastomer for the inner tube, a coating layer made from a biocompatible material such as hydrogel or silk biomaterials which can carry drug or bacteria resistant elements around the suture body is highly desired. An investigation on other biocompatible materials that can be directly integrated into the S^2^ suture components is essential before embarking on pre-clinical and clinical trials. In addition, the outer micro-coil will be upgraded by using a commercial suture wire as the main material. *Finally,* we also plan to refine, optimize, and combine the developed S^2^ sutures with our recently developed flexible surgical endoscopic robots for in-vivo experiments on a living animal. Our developed S^2^ sutures will also be a potential candidate for other surgical applications such as abdominal wound closure, hernia repair, sternal closure, or orthopedic procedures^49–52^.

In conclusion, we have introduced the design and fabrication of a new class of smart surgical sutures that can be automatically tightened and secure the tissue after making stitches. Ex-vivo experiments have been successfully conducted to demonstrate the new suture capabilities for perforation closure and tissue folding. We believe that this proof-of-concept study will inspire more related improvement and development and benefit the medical research community.

## Methods

### Material selection and fabrication process of the smart sutures

The micro-coils used for all smart sutures are stainless-steel extension spring coils (Asahi Intecc Co., Ltd., Japan). The microtubules are silicone tubing (Saint-Gobain S.A., France). *Firstly,* a microtubule is inserted into a micro-coil to form a STAM with an initial length *l*_*i*_. One end of the STAM is blocked while the other end is connected to a fluid source via a soft fluid transmission tube. Anchors are attached to both ends of the STAM. *Secondly,* the STAM is pressurized to the desired elongation ε with corresponding new length *l*_*p*_ (*l*_*p*_ > *l*_*i*_). While input pressure from the fluid source to the STAM is maintained, the soft fluid transmission tube is tied using an overhand knot to lock the hydraulic pressure inside the STAM. The other end of STAM is connected to a suture tip and a surgical needle to form the S^2^ suture-anchor. *Thirdly,* the S^2^ suture-anchor punctures through the tissue to form surgical stitches. *Fourthly,* the pressure locking mechanism is cut by a surgical knife or scissors to release the hydraulic pressure which will shorten the STAM and automatically deploy the anchors to tighten the tissue. The same procedure is also applied to the other types of anchors (lantern and sawtooth). It is noted that two ends of each sawtooth anchor are required to adhere to the micro-coil when the STAM is in the elongated state in order to achieve the bending motion afterward. The 3D printed anchors are made from polylactic acid (PLA + , Shenzhen Esun Industrial Co., Ltd., China) via a 3D printer (Ultimaker S3, Ultimaker B.V., Netherlands). The lantern and sawtooth anchors are manually fabricated from polyolefin tubes (heat shrink medical tubing, Nordson Medical, USA). The pressure locking mechanism (type sPLM) is a soft rubber tubing (McMaster-Carr Supply Co., USA). The suture tip is a 3D printed cone made from PLA + . The surgical needle is a nylon monofilament curved needle (Surgical Specialties Corporation, USA). All three S^2^ suture-anchor prototypes were pressurized to 50% of elongation (with the corresponding length *l*_*p*_ = 105 mm).

### Testing setup for the smart suture characterization

The testing platform consisted of a motorized linear slider (Zaber, Canada) to provide input volume (distilled water) and pressure from a fluid source (medical syringe, BD Biosciences, Canada) to a STAM specimen (Fig. 4A). A pressure sensor (Honeywell, USA) was located right after the syringe outlet to measure input pressure. The specimen distal end was connected to an encoder (US Digital, USA) to measure output displacement. Figure 4B shows the experimental setup to establish the STAM elongation-force relationship. A load cell (Futek, USA) was used to continuously record the tension force. For the burst pressure characterization, the sPLM (soft tube PLM) was similar to that of Fig. 4A. We supplied hydraulic fluid (distilled water) to a soft tube, tied a knot and then connected it to the syringe outlet. Subsequently, the motorized linear slider pumped pressure to the soft tube until it burst, while the pressure sensor was continuously logging real-time pressure data.

### Perforation closure with the S^2^ suture-knots

Each prototype was directly connected to a surgical needle at one end of the STAM and the other end was connected to a hydraulic syringe via a miniature fluid transmission tube. The smart sutures were then elongated to 100% elongation (for the STAM with OD1.49 mm) and 60% elongation (for STAM with OD0.8 mm). A long perforation on a fresh porcine colon was created, then continuous stitches were performed around the wound, creating a loose knot (Figs. 8A,B). The fluid pressure was then released to shorten the STAM, which then induced self-tightening of the knot. Unlike experiments for the S^2^ suture-anchors (Fig. 7) where the pressure locking mechanism was cut to rapidly reduce the hydraulic pressure, the hydraulic pressure was controlled via a miniature syringe in order to observe the dynamic motion of knot tying in real-time.

## Supplementary Information

Supplementary Video 1.

Supplementary Information 1.
