# Supplementary material for: Smart surgical sutures using soft artificial muscles
Source: Sci Rep. 2021 Nov 17;11:22420. doi: 10.1038/s41598-021-01910-2 (PMC8599709; doi:10.1038/s41598-021-01910-2)
Supplement: Supplementary file 2 — Supplementary Information 1. [file 41598_2021_1910_MOESM2_ESM.docx]

**Video Tittle**: Performance of Smart Surgical Sutures for Wound Closure

**Legend**: This video introduces different structures of the smart surgical sutures and their use for different surgical applications.
